# Personal

**Published:** 1896-07

**Authors:** 


### Personal.

---

Dr. F. Hubbell Mills, of Buffalo, has removed from 160 Franklin street to 83 West Mohawk street. He also has an office in room 416, Ellicott Square. Hours at his residence: 8-9 a. m., 1-3 and 8 p. m. At Ellicott Square, 9-11 a. m., 4-6 and 7-8 p. m.

---

Dr. Edgar A. Forsyth, of Buffalo, has removed from 159 Glenwood avenue to 64 West Huron street, corner Franklin. Practice limited to diseases of the nose and throat. Hours: 9 a. m. to 1 p. m. and by appointment.

Dr. A. J. Colton, of Buffalo, has removed from 151 East Ferry street to 27 East Ferry street, corner Otis place. Office hours: 8 to 9 A. M., 1 to 3 and 7 to 8 P. M.

